# Efficacy and tolerability of intravenous methylergonovine in migraine female patients attending the emergency department: a pilot open-label study

**DOI:** 10.1186/1746-160X-5-21

**Published:** 2009-11-08

**Authors:** Alfredo I Niño-Maldonado, Gary Caballero-García, Wilfrido Mercado-Bochero, Fernando Rico-Villademoros, Elena P Calandre

**Affiliations:** 1 Instituto de los Seguros Sociales, Universidad del Valle, Cali, Colombia; 2 Instituto de los Seguros Sociales, Universidad de Cartagena, Cartegena, Colombia; 3 Clínica La Milagrosa, Universidad Libre, Bogota, Colombia; 4 Instituto de Neurociencias, Universidad de Granada, Granada, España

## Abstract

**Background:**

Methylergonovine is an ergot alkaloid widely used in postpartum women. It is also an active metabolite of methysergide and previous studies suggest that it could be effective against refractory headache and cluster headache. The purpose of the present study was to assess the potential therapeutic effectiveness of methylergonovine in the emergency treatment of severe migraine.

**Methods:**

One hundred and twenty five female patients with migraine attending the emergency department received 0.15 mg of methylergonovine intravenously. Pain intensity, heart rate, blood pressure, and methylergonovine side effects were checked 5, 10, 15, 30 and 60 minutes after drug administration. An additional 0.075 mg dose of methylergonovine was administered to those patients who did not experienced relevant pain relief 15 minutes after dosing.

**Results:**

Pain intensity decreased markedly from the first minutes after dosing, the 74.4% of patients being pain free at 60 minutes. Only seven patients required an additional dose of methylergonovine. Nausea and vomiting were the most relevant side effects related with methylergonovine administration (84% of patients). A substantial decrease (10 to 25 mmHg) in systolic blood pressure values was observed in 56% of the patients. A significant correlation (p < 0.0001) was found between the decrease in pain intensity and the reduction of systolic blood pressure.

**Conclusion:**

Although limited by the non-controlled design of the study, our data suggest that intravenous methylergonovine can be an effective and safe drug in the management of severe migraine attacks in the emergency room.

## Background

The treatment of migraine attacks in the emergency room includes the use of several kind of drugs, usually administered by parenteral route, namely non-steroidal antiinflamatory drugs (NSAIDs), opioids, glucocorticoids, neuroleptics, ergot alkaloids and triptans [1]. Among ergot alkaloids, dihydroergotamine, available as intranasal, subcutaneous, intramuscular or intravenous formulations, has been shown to be effective and well tolerated [2], and its intravenous administration has been advocated in the management of status migrainosus [3]. However, dihydroergotamine, which is a cheap and effective drug for the acute therapy of migraine attacks, is only available in few countries.

Methylergonovine, also called methylergometrine, is another ergot alkaloid, a semisynthetic derivative of LSD, widely used in the postpartum for its uterotonic properties. Additionally, methylergonovine is also a major active metabolite of methysergide whose area under the curve after oral administration of methysergide is substantially higher than the one of the parent drug [4]. Methylergonovine has been postulated to play an important role in the methysergide therapeutic efficacy in migraine [5]. Earlier publications have shown that oral administration of methylergonovine could be effective in the control of the drug induced refractory headache [6] as well as in the management of cluster headache [7].

The purpose of the present study was to evaluate the potential effectiveness of intravenous methylergonovine in the acute treatment of severe migraine previously refractory to non-steroidal analgesic drugs (NSAID).

## Methods

The study was done in the Emergency Departments of two hospitals of the district of Santa Marta, in Colombia. Inclusion criteria were female adult patients experiencing migraine with or without aura according to the IHS diagnostic criteria [8], and suffering an attack of severe intensity (≥8 in a 10 cm Visual Analog Scale) who attended the emergency room because the pain had not improved with their usual home treatment. Every patient provided written informed consent to participate in the study.

Women experiencing current or previous cardiovascular disease, morbid obesity, epilepsy, serious (DSM IV Axis I) psychiatric disease, known intolerance to ergot alkaloids, pregnant or lactating were excluded from the study, as well as those who have received ergotamine or triptans in the previous twenty four hours.

Pain intensity was assessed by means of a Visual Analog Scale (VAS) just before drug administration and 5, 10, 15, 30 and 60 minutes after. Arterial blood pressure and heart frequency were measured, and emergent drug adverse reactions were recorded at the same time points. Methylergonovine was administered intravenously diluted in 5 ml of saline at an initial dosage of 0.15 mg. If pain intensity was still above 5 in pain VAS, an additional dose of 0.075 mg of methylergonovine was administered 15 minutes after the first one. Analgesic drug intake before admission was recorded. In fourteen (11%) patients, randomly selected, continuous monitoring of the ECG was performed during the all the observation period.

Data were analyzed by means of GraphPad Prism version 5.0 (GraphPad Software, San Diego, California, USA, http://www.graphpad.com.). Repeated measures analysis of variance was applied to analyze the evolution of pain severity, systolic and diastolic blood pressure, and heart rate. Effects sizes were caluculated according to the Cohen's formula and considered large when equal or higher than 0.80 [9]. Comparison between data from patients requiring one and two doses of methylergonovine was done with the Student's *t *test for unpaired data. The Spearmen correlation coefficients were used to evaluate the potential relationship between systolic blood pressure and pain intensity.

## Results

One hundred and twenty five patients participated in the study. One hundred and eleven (88.8%) suffered migraine without aura and 14 (11.2%) migraine with aura. Their age ranged from 25 to 45 years (45 ± 7). All patients reported photophobia, phonophobia, nausea and vomiting at baseline. Previous analgesic intake included paracetamol (55%), aspirin (16%), ibuprofen (16%), metamizole (9,6%), diclofenac (8%), and combined analgesics (6.4%).

**Figure 1:**
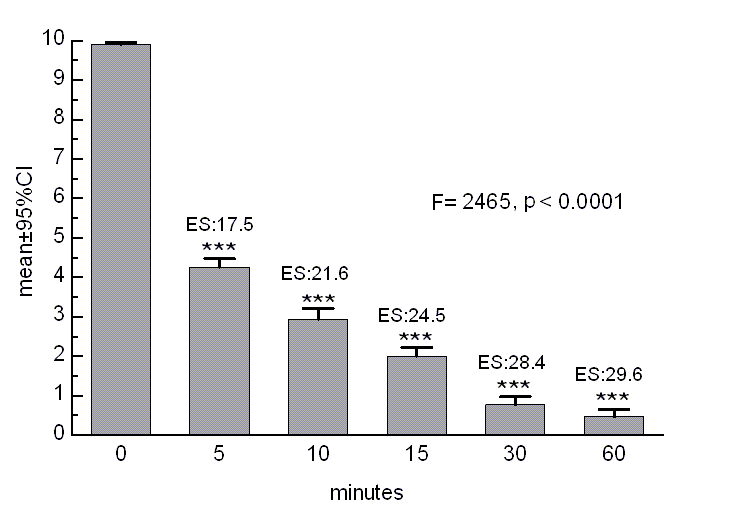
Pain intensity over the monitored time period (***: p < 0.0001 in relation to baseline; ES: effect size).

Pain intensity subsided quickly after methylergonovine administration until reaching a mean value of 0.46 ± 1.1 60 minutes after dosing. As it can be seen in Figure 1, the decrease in pain severity was statistically significant and clinically relevant already 5 minutes after drug injection.

**Figure 2:**
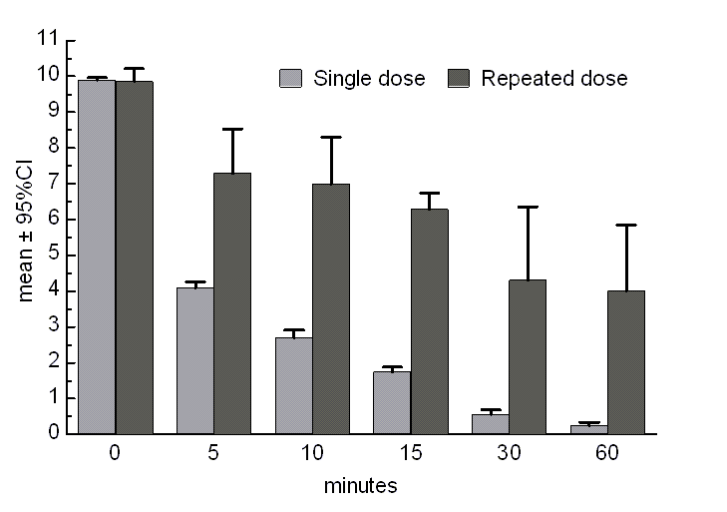
**Pain intensity over the monitored time period in patients receiving one (grey bars) or two doses (black bars) of methylergonovine**.

Only seven (5.6%) patients required a second additional dose of methylergonovine 15 minutes after the initial one. As it is shown in Figure 2, although baseline pain severity was similar among patients who needed only one dose of methylergonovine and those who needed two doses (9.91 ± 0.32 vs 9.86 ± 0.38, not significant), final pain intensity was significantly higher at endpoint among the later group (0.25 ± 0.51 vs 4 ± 2, p < 0.0001).

Complete disappearance of pain was reached by 66 (52.8%) patients 30 minutes after dosing and by 93 (74.4%) patients 60 minutes after dosing. Only one of the patients requiring an additional dose of methylergonovine was pain free at 60 minutes.

**Figure 3:**
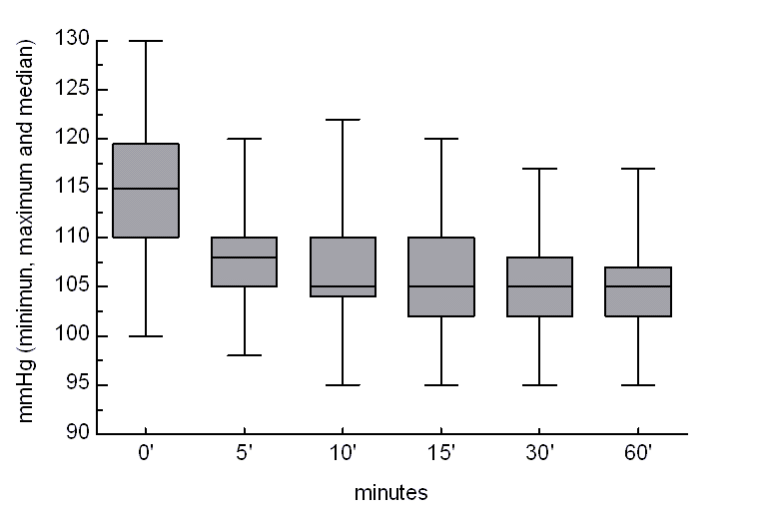
**Evolution of systolic blood pressure values over the monitored period**.

**Figure 4:**
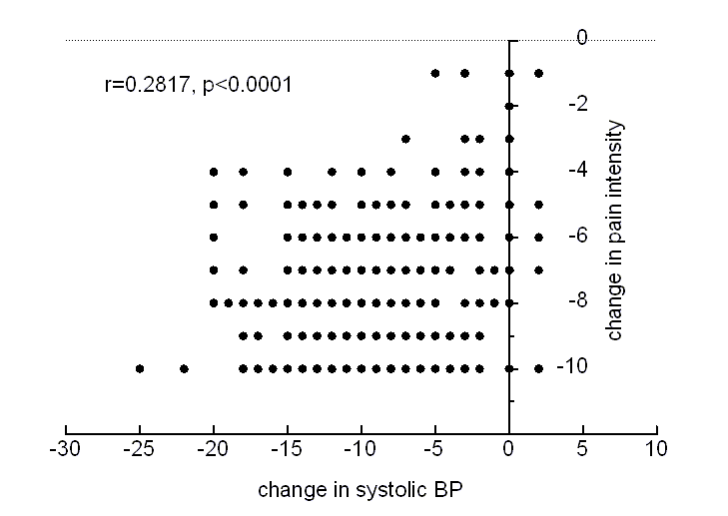
**Correlation among individual changes in pain intensity and systolic blood pressure**.

Table 1 shows arterial blood pressure and heart frequency data. Neither blood pressure nor heart frequency increased in any patient. On the contrary, a reduction in mean baseline systolic blood pressure values was observed along the time (Figure 3). A significant correlation(r = 0.2817, p < 0.0001) was found between the change in systolic blood pressure and the decrease in pain intensity (Figure 4). No electrocardiographic abnormalities were found in any of the patients whose ECG was monitored.

**Table 1:** Range, mean and SD values of blood pressure and heart rate during the monitored period

	Systolic BP	Diastolic BP	Heart rate
Baseline	(100-130) 114.7-6.47	(55-80) 61.5 ± 3.95	(60-88) 72.8 ± 4.9

5 minutes	(98-120) 107.3-4.78	(55-75) 60.9 ± 3.83	(60-82) 69.5 ± 5.6

10 minutes	(95-122) 106.6 ± 4.67	(51-75) 60.4 ± 3.9	(55-82) 68.5 ± 5.5

15 minutes	(95-120) 105.7 ± 4.86	(51-75) 59.8 ± 3.9	(55-82) 67.8 ± 5.3

30 minutes	(95-117) 104.8 ± 4.46	(54-76) 59.6 ± 3.7	(53-80) 67.5 ± 5.1

60 minutes	(95-117) 104.5 ± 4.43	(54-75) 59.7 ± 3.4	(53-80) 67.4 ± 4.8
P	< 0.0001	< 0.0001	< 0.0001

Nausea (84.8%) and vomiting (84%) were the most frequent side effects related with methylergonovine administration, followed by fatigue (34.4%), diaphoresis (12%), and dizziness (11.2%). Seventy two (57.6%) patients reported perceptive alterations, mainly sensation to be floating, sensation of peacefulness, and feelings of pleasant relaxation.

## Discussion

Intravenous methylergonovine originated a quick and strong relief of pain in our patients. The degree of improvement, either considering the mean VAS score 60 minutes after dosing or the percentage of patients experiencing total pain relief, was similar to the improvement described with the use of intravenous prochlorperazine, a conventional antipsychotic, in several randomized clinical trials [10-12].

It is difficult to compare our data with those published for dihydoergotamine because, with only one exception in which it was found to be less effective than chlopromazine [13], this drug has been administered either by intramuscular route compared with subcutaneous sumatriptan, or associated with an antiemetic, usually metoclopramide which is known to exert an antimigraine activity of its own [14,15]. A recent systematic review of trials performed with dihydroergotamine concluded that, associated with an antiemetic, it seems to be as effective as the other active comparators, but underlined that the methodology and scientific quality of the trials was highly variable [15].

The fact that only one of the seven patients who received an additional dose of methylergonovine achieved complete pain relief, suggests that little or no drug benefit can be expected in initially nonresponder patients.

Most frequent methylergonovine side effect is hypertension, hypotension having been described less frequently [16]. However, none of our patients experienced an increase in blood pressure. On the contrary, a clinically relevant decrease in systolic blood pressure values, ranging from 10 to 25 mmHg without relevant changes in diastolic blood pressure, was observed in 70 (56%) patients 60 minutes after drug administration. As transient hypertension has been described associated with migraine attacks [17], we thought that the observed reduction in systolic blood pressure could be related with pain relief. The fact that a significant correlation was found between individual changes in systolic blood pressure and pain relief (Figure 4) supports this explanation.

Nausea and vomiting have been reported to occur occasionally following methylergonovine administration [16]. In our sample these were the most frequent adverse event reported by our patients. However, as all of them, suffered these symptoms associated to their migraine attack at hospital arrival, it is difficult to ascertain if nausea and vomiting were due to the effect of the drug or to the underlying disease.

In our study men with migraine were excluded because methylergonovine, as an uterotonic drug, is basically used in women, and data concerning its pharmacological activity in men are lacking. Methylergonovine pharmacokinetics has been studied in women and in men: no differences between them were found when the drug was administered intravenously and, after oral administration, the wide interindividual variation in pharmacokinetic parameters did not allowed to compare adequately both groups [18]. Additionally, pharmacodynamic differences could exist and influence both drug efficacy and tolerability, for instance at cardiovascular level.

The main limitation of our study is its non-controlled design due to the difficulties to carry out a controlled randomized clinical trial in Colombia, where this kind of studies are not easily performed if they are not sponsored by a international pharmaceutical company, mainly due to financial reasons. However, we think that the sample size of our study, the pronounced effect over pain, and the low interindividual variability of the data support the consistency of our results.

An additional limitation is the fact that patients were dismissed from the hospital 30 minutes after the last evaluation, and were not followed later to assess headache recurrence. Methylergonovine half-life is short - 1.94 ± 0.34 hours after i.v. administration in women [18] and recurrence rate should have been assessed.

There is a need for effective and low cost drugs for the acute treatment of severe migraine and status migrainosus. Sumatriptan is a very effective but expensive drug. Opioids are scarcely effective and most headache specialists advise against their use [19]. Although dexamethasone seems to be effective in reducing migraine attack's recurrence, its efficacy to decrease attack's pain is similar to placebo [20]. Some conventional neuroleptics, as prochlorperazine and chlorpromazine, have been used by parenteral route in the acute management of migraine [21], being akathisia is the most common side effect associated with intravenous prochlorperazine and postural hypotension with chlorpromazine. Dihydroergotamine seems to be especially effective only when associated to an antiemetic and, on the other hand, is not available worldwide. Methylergonovine is available in many countries, both in oral and parenteral formulations, and has a very low acquisition cost, a factor which is very important, especially for developing countries. Randomized controlled trials in patients of both sexes seem to be warranted in order to ascertain its potential role in the acute management of migraine.

## Conclusion

Despite the above mentioned limitations, we think that our results suggest that intravenous methylergonovine, at least in the medium dose used in this study, can be an effective and safe drug in the emergency management of severe refractory migraine, and in an environment with limited access to health resources could be an alternative to triptans.

## Competing interests

The authors declare that they have no competing interests.

## Authors' contributions

AINM, GCG and WMB diagnosed and treated patients. EPC and FR participated in the design of the study and drafted the manuscript. All authors read and approved the manuscript's final version.

## References

[B1] Martelletti P Farinelli I Steiner TJ Acute migraine in the Emergency Department: extending European principles of management Intern Emerg Med 2008 3 Suppl 1 S17 S24 10.1007/s11739-008-0188-1 18785015

[B2] Saper JR Silberstein S Pharmacology of dihydroergotamine and evidence for efficacy and safety in migraine Headache 2006 46 Suppl 4 S171 S181 10.1111/j.1526-4610.2006.00601.x 17078849

[B3] Saper JR Silberstein S Dodick D Rapoport A DHE in the pharmacotherapy of migraine: potential for a larger role Headache 2006 46 Suppl 4 S212 S220 10.1111/j.1526-4610.2006.00605.x 17078853

[B4] Bredberg U Eyjolfsdottir GS Paalzow L Tfelt-Hansen P Tfelt-Hansen V Pharmacokinetics of methysergide and its metabolite methylergometrine in man Eur J Clin Pharmacol 1986 30 75 77 10.1007/BF00614199 3709634

[B5] Müller-Schweinitzer E Taparelli C Methylergometrine, an active metabolite of methysergide Cephalalgia 1986 6 35 41 10.1046/j.1468-2982.1986.0601035.x 3698092

[B6] Graff-Radford SB Bittar GT The use of methylergonovine (Methergin^(r)^) in the initial control of drug induced refractory headache Headache 1993 33 390 393 10.1111/j.1526-4610.1993.hed3307390.x 8376101

[B7] Mueller L Gallagher RM Ciervo CA Methylergonovine maleate as a cluster headache prophylactic: a study and review Headache 1997 37 437 442 10.1046/j.1526-4610.1997.3707437.x 9277027

[B8] Headache Classification Committee of the International Headache Society Classification and diagnostic criteria for headache disorders, cranial neuralgias and facial pain Cephalalgia 1988 8 Suppl 7 1 96 3048700

[B9] Cohen J Statistical power analysis for the behavioral sciences 1988 2 Hillsdale, New Jersey: Laurence Earlsbaum Associates

[B10] Jones J Sklar D Dougherty J White W Randomized double-blind trial of intravenous prochlorperazine for the treatment of acute headache JAMA 1989 261 1174 1176 10.1001/jama.261.8.1174 2915441

[B11] Coppolo M Yealy DM Leibold RA Randomized, placebo-controlled evaluation of prochlorperazine versus metoclopramide for emergency department treatment of migraine headache Ann Emerg Med 1995 26 541 546 10.1016/S0196-0644(95)70001-3 7486359

[B12] Seim MB March JA Dunn KA Intravenous ketorolac vs intravenous prochlorperazine for the treatment of migraine headaches Acad Emerg Med 1998 5 573 576 9660282 10.1111/j.1553-2712.1998.tb02463.x

[B13] Bell R Montoya D Shuaib A Lee MA A comparative trial of three agents in the treatment of acute migraine headache Ann Emerg Med 1990 19 1079 1082 10.1016/S0196-0644(05)81507-0 2221511

[B14] Colman I Brown MD Innes GD Grafstein E Roberts TE Rowe BH Parenteral metoclopramide for acute migraine: meta-analysis of randomised controlled trials BMJ 2004 329 1369 1373 15550401 10.1136/bmj.38281.595718.7C PMC535449

[B15] Colman I Brown MD Innes GD Grafstein E Roberts TE Rowe BH Parenteral dihydroergotamine for acute migraine headache: a systematic review of the literature Ann Emerg Med 2005 45 393 401 10.1016/j.annemergmed.2004.07.430 15795718

[B16] Methylergonovine package insert http://www.accessdata.fda.gov/drugsatfda_docs/label/2007/006035s075lbl.pdf (accesed October 2009)

[B17] Mathew NT Migraine and hypertension Cephalalgia 1999 19 Suppl 25 17 19 10668113 10.1177/0333102499019s2504

[B18] De Groot AN Vree TB Hekster YA Van Den Biggelaar-Martea M Van Dongen PW Van Roosmalen J Comparison of the bioavailability and pharmacokinetics of oral methylergometrine in men and women Int J Clin Pharmacol Ther 1995 33 328 332 7582383

[B19] Green MW The emergency management of headaches The Neurologist 2003 9 93 98 10.1097/01.nrl.0000051443.03160.72 12808371

[B20] Colman I Friedman BW Brown MD Innes GD Grafstein E Roberts TE Rowe BH Parenteral dexamethasone for acute severe migraine headache: meta-analysis of randomised controlled trials for preventing recurrence BMJ 2008 336 1359 1361 18541610 10.1136/bmj.39566.806725.BE PMC2427093

[B21] Hua C Young WB Silberstein SD Neuroleptics in headache Headache 2005 45 358 371 10.1111/j.1526-4610.2005.05074.x 15836574

